# Mechanism of human α3β GlyR modulation in inflammatory pain and 2, 6-DTBP interaction

**DOI:** 10.21203/rs.3.rs-4402878/v1

**Published:** 2024-08-07

**Authors:** Weiwei Wang, Xiaofen Liu

**Affiliations:** University of Texas Southwestern Medical Center; University of Texas Southwestern Medical Center

## Abstract

α3β glycine receptor (GlyR) is a subtype of the GlyRs that belongs to the Cys-loop receptor superfamily. It is a target for non-psychoactive pain control drug development due to its high expression in the spinal dorsal horn and indispensable roles in pain sensation. α3β GlyR activity is inhibited by a phosphorylation in the large internal M3/M4 loop of α3 through the prostaglandin E2 (PGE_2_) pathway, which can be reverted by a small molecule analgesic, 2, 6-DTBP. However, the mechanism of regulation by phosphorylation or 2, 6-DTBP is unknown. Here we show M3/M4 loop compaction through phosphorylation and 2, 6-DTBP binding, which in turn changes the local environment and rearranges ion conduction pore conformation to modulate α3β GlyR activity. We resolved glycine-bound structures of α3β GlyR with and without phosphorylation, as well as in the presence of 2, 6-DTBP and found no change in functional states upon phosphorylation, but transition to an asymmetric super open pore by 2, 6-DTBP binding. Single-molecule Forster resonance energy transfer (smFRET) experiment shows compaction of M3/M4 loop towards the pore upon phosphorylation, and further compaction by 2, 6-DTBP. Our results reveal a localized interaction model where M3/M4 loop modulate GlyR function through physical proximation. This regulation mechanism should inform on pain medication development targeting GlyRs. Our strategy allowed investigation of how post-translational modification of an unstructured loop modulate channel conduction, which we anticipate will be applicable to intrinsically disordered loops ubiquitously found in ion channels.

## Introduction

GlyRs are members of the ionotropic pentameric Cys-loop receptor superfamily that mediates inhibitory neurotransmission in the central nervous system^1,2^. In adult animals, heteromeric GlyRs consisting of both α (α1-α4) and β subunits with a 4α:1β stoichiometry are the dominant form^3–5^. Dysfunction of GlyRs in the brain is associated with multiple neurological disorders, including Alzheimer’s disease, schizophrenia, epilepsy and autism^6–11^. In the spinal cord, GlyRs are highly expressed and play major roles in inhibitory neurotransmission, with subtype-specific localization and physiology^1,2,12–14^. The α1β/α2β subtype of GlyRs are found throughout the spinal cord and governs locomotive functions and harbor mutagenesis sites that cause the congenital disease hyperekplexia^15–19^, while the α3β GlyR is found highly concentrated in superficial layers of the dorsal horn, where sensory signals are processed before entering the brain^1,2,20,21^.

**Figure 1:**
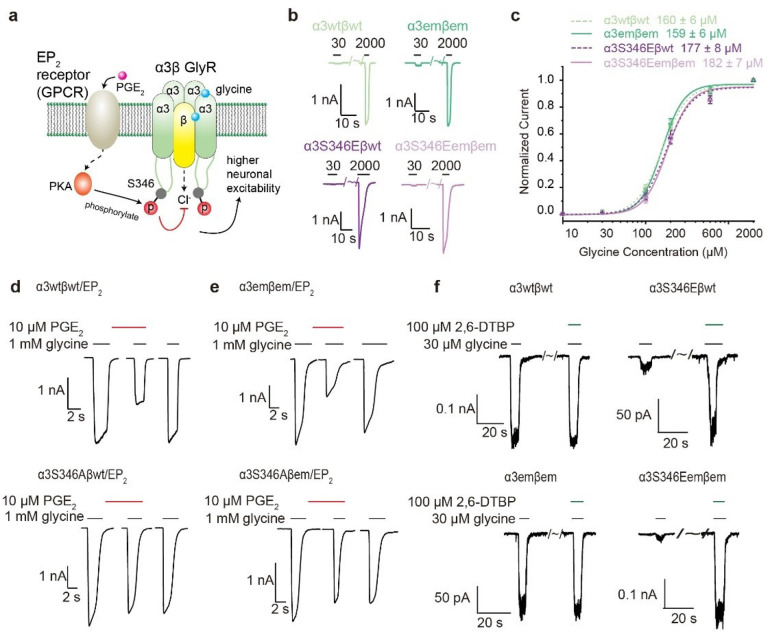
Functional characterization of human α3β GlyR. **a,** The effect of PGE_2_ on glycinergic signaling. PGE_2_ activates EP_2_ receptors which in turn phosphorylate α3 subunits in a PKA-dependent manner to inhibit Cl^−^ flux through α3β GlyR. **b,** Typical glycine response of α3wtβwt, α3emβem, α3S346Eβwt and 3S346Eemβem at 30 μM and 2 mM glycine concentration. **c,** Dose response with Hill fits (lines). Data are represented as mean ± S.E.M. (n=7–11 cells). **d,** Representative the effect of 10 μM PGE_2_ on glycine-evoked current traces in HEK293T cells co-transfected with the α3wtβwt and EP_2_ receptor (top), and after disruption of the PKA consensus sequence by introducing the S346A mutation(down). **e,** Representative the effect of 10 μM PGE_2_ on glycine-evoked current traces in HEK293T cells co-transfected with the α3emβem and EP_2_ receptor (top), and after introducing the S346A mutation(down). **f,** Representative traces of glycine-induced whole-cell currents recorded from HEK293T cells expressing α3wtβwt, α3emβem, α3S346Eβwt and 3S346Eemβem GlyRs in the absence or presence of 100 μM 2, 6-DTBP.

Chronic pain is a very common problem that affects over 20% individuals in the US and world-wide^22–24^. α3β GlyR is an essential player in inflammation related hyperalgesia (increased perception of pain)^25,26^ and a promising drug target for non-psychoactive pain control^26–29^. Unlike most GlyR-related disordered where loss of function mutations are the major culprit, α3β GlyR modulates pain sensation through post-translational modification in the large internal M3-M4 loop (~ 80 amino acid residues)^25,30^. This loop is responsible for post-synaptic localization and involved in gating property modulation of GlyRs^31–37^. Phosphorylation of α3S346 shows one of the most well characterized functional and pharmacological roles of M3-M4 loop^25,38–41^. Prostaglandin E2 (PGE_2_) produced under inflammatory conditions activates the Prostaglandin E2 receptor 2 (EP_2_), which in turn results in protein kinase A (PKA)-dependent phosphorylation of residue S346 in the large internal M3/M4 loop of the GlyR α3 subunit, reducing Cl^−^ conduction^25,41,42^ (Fig. 1 a). Unfortunately, partly due to the lack of structural information, how a distal loop phosphorylation (~ 50 aa from pore) modulates ion conduction in α3β GlyR is unclear^37,38,41^.

A derivative of the general anesthetic propofol, 2, 6-di-*tert*-butylphenol(2, 6-DTBP), that does not bind to γ-Aminobutyric Acid type A (GABA_A_) and is non-psychoactive^43^, potentiates α3β GlyR and alleviates hyperalgesia in a mouse model of neuropathic pain^39,44^. The positive modulating effect of 2, 6-DTBP appears to be dependent on the phosphorylation of α3β GlyR^39,41^, and related to an aromatic amino-acid residue near the intracellular opening of the conduction pore^45^. Based on these observations, 2, 6-DTBP seem to represent a promising pain control that specifically potentiates α3β GlyR without unwanted side effects. However, the working mechanism of 2, 6-DTBP remain mysterious.

The absence of α3β GlyR structure, as well as the lack of understanding in the conformation of the large internal M3/M4 loop, has severely limited our understanding of α3β GlyR regulation in pain conditions, and how new types of pain control molecules may be developed. Here, we report near-atomic resolution structures of the human heteromeric α3β GlyR bound with glycine (αβ-gly GlyR) in both digitonin detergent and nanodiscs, showing minimal differences between them. We also determined structures of phosphorylation mimetic α3S346Eβ-gly GlyR, and its complex with 2, 6-DTBP, α3S346Eβ-gly/2, 6-DTBP GlyR, where we observed asymmetrical pore rearrangements upon phosphorylation and 2, 6-DTBP binding. We further characterized the M3/M4 loop conformation using single molecular fluorescence resonance energy transfer (smFRET) method, which revealed changes in loop conformation upon phosphorylation and 2, 6–DTBP binding. Combining structural, electrophysiological and smFRET observations, we propose a mechanism where phosphorylation and 2, 6-DTBP exert functional effects through altering M3/M4 loop conformation that leads to changes in local electrostatics and rearrangements in the TM. This mechanism helps in understanding the regulation of α3β GlyR in inflammatory pain, and possible pharmacological intervention. In addition, it provides a framework for understanding how M3/M4 loop regulate Cys-loop and other related receptors through post-translational modification.

## Results

### α3emβem GlyR recapitulates wild-type modulation in PGE2 pathway

We generated α3em through a small deletion in the M3/M4 loop without affecting the PKA consensus phosphorylation sequence (RESR, positions 344–347, Extended Data Fig. 1a). The S346E mutation, a good mimetic of phosphorylation^20,38,41^, was also made for both α3em and α3wt.

Co-expression of α3em and the βem that we reported previously (Extended Data Fig. 1b)^3,5,37^ allowed for the assembly of functional α3emβem GlyR, which showed much improved biochemical behavior compared to the wild-type (Extended Data Fig. 1c) while retaining indistinguishable glycine EC_50_ (~ 160 μM) and activation Hill slope (~ 3) (Fig. 1b, c, Extended Data Fig. 1f green and light green). S346E had marginal effect on glycine activation, increasing glycine EC_50_ of both α3emβem and α3wtβwt GlyR to ~ 180 μM without changing Hill slope (Fig. 1b, c, Extended Data Fig. 1f purple and light purple).

Ion conduction of α3emβem GlyR and α3wtβwt GlyR was similarly modulated through EP_2_ receptor activation. Application of 10 μM PGE_2_ to HEK293T cells co-expressing EP_2_ receptors reduced glycine-evoked currents by ~ 50% for both α3wtβwt and α3emβem GlyR (Fig. 1d, e upper panels). Mutation of the phosphorylation site, S346A, abolished this effect (Fig. 1d, e lower panels). Consistent with previous reports^39,43^, 2, 6-DTBP strongly potentiated glycine-induced currents for both α3wtβwt (3.7 ± 0.5 folds, n = 9) and α3emβem (3.2 ± 0.4 folds, n = 10) GlyR, in a phosphorylation (S346E)-dependent manner (Fig. 1f).

Taken together, α3emβem GlyR recapitulates functional properties of wild-type α3β GlyR in glycine-activation, PGE_2_-dependent modulation, as well as phosphorylation-dependent potentiation by 2, 6-DTBP.

### S346 phosphorylation and 2, 6-DTBP binding alter ion-conduction pore conformation

**Figure 2:**
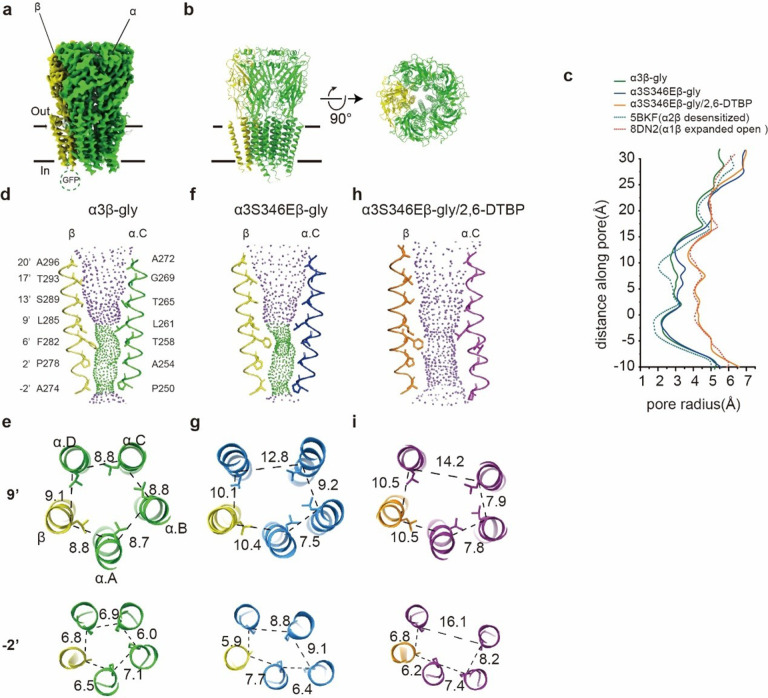
Conformational differences between α3β-gly, α3S346Eβ-gly, and α3S346E3-gly/2, 6-DTBP GlyRs. **a,** Side view of cryo-EM map of α3β GlyR in digitonin in presence of glycine. **d,** Side (left) and top-down (right) view of the atomic models. α subunits and β subunit are respectively colored in bright green and yellow. **c,** Plot of pore radii calculated by the HOLE program for the α3β-gly (green), α3S346Eβ-gly (blue), α3S346Eβ-gly/2, 6-DTBP (orange), α2β-gly (desensitized, PDB ID: 5BKF, green dashed line), and α1β-gly (expanded open, PDB ID:8DN2, red dashed line). **d, f, h,**Ion permeation pathways for α3β-gly(d), α3S346Eβ-gly(f), and α3S346Eβ-gly/2, 6-DTBP(h) GlyRs. M2 helices are shown as cartoon and the side chains of pore-lining residues as sticks. Purple, green, red spheres define radii of > 3.3 Å, 1.8–3.3 Å, and < 1.8 Å, respectively. **e, g, i,** Cross-sections of M2 helices at residues 9’ (top) and −2’ (bottom) for α3β-gly(e), α3S346Eβ-gly(g), and α3S346Eβ-gly/2, 6-DTBP(i) GlyRs with distances between neighboring Cα shown in Å.

To elucidate how phosphorylation at α3S346 and 2, 6-DTBP modulates glycine-elicited α3β GlyR currents, we resolved glycine-bound structures of α3emβem GlyR (α3β-gly, 3.8 Å, Extended Data Fig. 2a-e), phosphorylation mimic α3emS346Eβem GlyR (α3S346Eβ-gly, 3.7 Å, Extended Data Fig. 3a-e), and phosphorylation mimic α3emS346Eβem GlyR with 2, 6-DTBP (α3S346Eβ-gly/2, 6-DTBP, 3.6 Å, Extended Data Fig. 3f-j), in digitonin detergent. To identify structural changes arising from detergent/lipid mimetic biochemistry, we also resolved glycine-bound structure of α3emβem GlyR reconstituted in saposin nanodisc (α3β-gly (nanodisc), 3.8 Å, Extended Data Fig. 2f-j). All density maps allowed unambiguous model-building (Extended Data Fig. 2–4) and showed a 4:1 α3:β subunit stoichiometry (Fig. 2a, b), consistent with the stoichiometry in other two major human heteromeric GlyR types, α1β^5,46^ and α2β^3^, as well as a zebrafish analog α1β_B_^47^. The structures in digitonin and nanodisc were virtually identical throughout the extracellular (ECD) and transmembrane domains (TM) (Fig. 2d, e, Extended Data Fig. 5a-e, Extended Data Fig. 6a, c, d), with a RMSD of ~ 0.9 Å, suggesting that digitonin provides a suitable biochemical environment for characterizing GlyR structures.

The α3β-gly structures (in digitonin and nanodisc) showed a pore conformation typical of a desensitized GlyR channel^3,5,46,48–50^ (Fig. 2c, d). The gate at the 9’ position (α3: L261, β: L285) is in the open conformation, while a constriction (~ 2.1 Å radius) at −2’ was observed (Fig. 2c, d, e). A pseudo-5-fold symmetry was maintained throughout the ECD (Extended Data Fig. 6b, d) and TM.

The phosphorylation mimic structure, α3S346Eβ-gly had a pseudo-5-fold symmetric ECD (Extended Data Fig. 6b, e), but asymmetric TMD conformation as has been widely observed in heteromeric GlyRs^3,5,46,47^ (Fig. 2c, f, g). The 9’ gate remained open, with an asymmetrically constricted – 2’ position (2.1 Å radius). Radii along the pore are very similar to those of α3β-gly structure, suggesting a desensitized functional state. Clearly, phosphorylation at S346 affected TM conformation despite it being situated in the long and unstructured M3/M4 loop.

2, 6-DTBP induced an asymmetric expanded-open pore conformation in the phosphorylation mimic GlyR (α3S346Eβ-gly/2, 6-DTBP) structure (Fig. 2c, h, i), while maintaining a pseudo-5-fold symmetric ECD (Extended Data Fig. 6d, f). Both the 9’ and the – 2’ positions dilated asymmetrically, with a minimum pore radius of ~ 3.9 Å at the - 2’ position, which is too large for the expected open GlyR pore based on electrophysiology measurements^49,51^. The pore geometry is very similar to previously reported expanded/super open states^5,48,50^. Although 2, 6-DTBP led to dramatic changes in channel conformation, its cryo-EM density cannot be unambiguously identified. In addition, the density for M3/M4 loop is missing in all the structures.

### Widening of α3: α3 TM interface underlie pore conformational change

The structures of α3β-gly, α3S346Eβ-gly and α3S346Eβ-gly/2, 6-DTBP are essentially identical in the ECD, showing pseudo-5-fold symmetry in the glycine-bound conformation with capped C-loops (Extended Data Fig. 6g, h). Differences in the ion conduction pore apparently arose from re-arrangements in the TMs.

**Figure 3:**
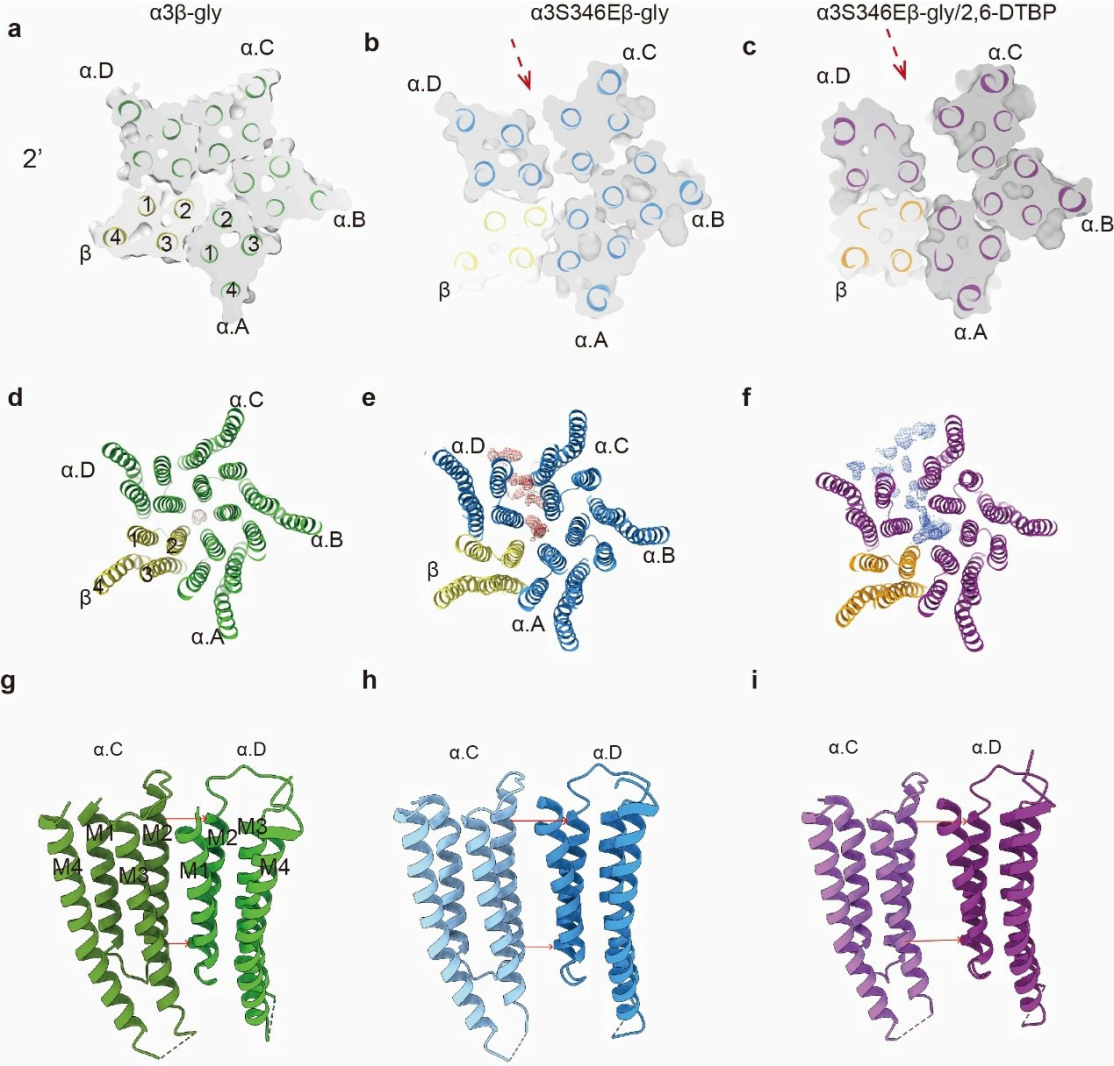
TMD of α3β and α3S346Eβ GlyRs conformational differences and 2, 6-DTBP induces α3S346Eβ GlyR conformational changes **a, b, c,** Z-slice in the TMD at 2’ for α3β-gly (a), α3S346Eβ-gly (b) and α3S346Eβ-gly/2, 6-DTBP(c). **d, e, f,** Top-down view non-protein densities of α3β-gly (d), α3S346Eβ-gly (e) and α3S346Eβ-gly/2, 6-DTBP (f) models contoured at 5 RMSD. Non-protein densities at widened subunit interfaces, and those in conduction pathways are colored in brown for α3β-gly (d), red for α3S346Eβ-gly (e) and blue for α3S346Eβ-gly/2, 6-DTBP (f), respectively. **g, h, i,** The cartoon representation of the TMD interfaces between α.C and α. D subunits for α3β-gly (g), α3S346Eβ-gly (h), α3S346Eβ-gly/2, 6-DTBP (i) GlyRs. Red arrow indicates distance between adjacent α subunits at −2’ (down) and 19’(top).

Changes of distances in the TM between neighboring α3 subunits contribute to differences in pore conformations. The TM of α3β-gly GlyR, in the desensitized state, showed 5-fold pseudo-symmetry and tight packing (Fig. 3a, g). Non-protein densities were only observed in the conduction pore, and some of which likely correspond to bound ions (Fig. 3d). In contrast, the distance between two α3 subunits (the one next to the α3: β interface) widened in the α3S346Eβ-gly GlyR structure (Fig. 3b). The same α3: α3 interface widened more dramatically in the α3S346Eβ-gly/2, 6-DTBP GlyR (Fig. 3c) structure. A similar increase in inter-α subunit distance in the TM has been observed before in α1β GlyR and found relevant to channel opening^5^. α3 subunits showed similar positive charges in the TM interface (Extended Data Fig. 5j, k) as α1, likely resulting in electrostatic repulsion and promotion of the widening interface.

Since only 1 out of 5 TM interfaces significantly widened, 5-fold pseudo-symmetry was broken for both α3S346Eβ-gly and α3S346Eβ-gly/2, 6-DTBP GlyR structures. In addition to the ion conduction pores, extra densities were found in the widened inter-α TM gaps (Fig. 3e, f). These densities may represent substances that stabilize the asymmetric TM conformation with larger spaces between one α3: α3 subunit interface (Fig. 3h, i).

### α3 M3/M4 loop approximates the pore upon phosphorylation and 2, 6-DTBP binding

**Figure 4:**
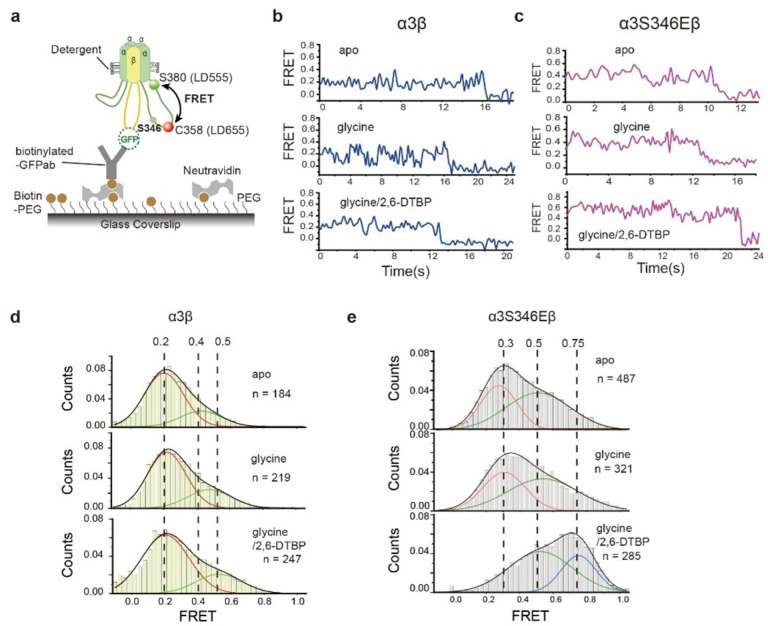
α3S346E mutation and 2, 6-DTBP modulate internal conformation of the M3/M4 loop in α3S346Eβ GlyR **a,** Schematic of single-molecule FRET experiments for measuring the FRET value changes of the internal M3/M4 loop induced by α3S346E mutation and 2, 6-DTBP. **b, c,** Representative single-molecule FRET time traces of α3β(b) and α3S346Eβ(c) GlyRs in apo (top), in presence of glycine (middle) and in presence of glycine and 2, 6-DTBP (bottom). **d, e,** Histograms of smFRET efficiency values from single-molecules traces for α3β(d) and α3S346Eβ (e) GlyRs in apo (top), in presence of glycine (middle) and in presence of glycine and 2, 6-DTBP (bottom). Two smFRET distributions indicated by curves of Gaussian fitting and the sum of all distributions are shown as black lines.

To understand how phosphorylation at S346, which is ~140 Å from the pore if M3/M4 loop is fully extended, affect ion conduction, we used single-molecule Forster resonance energy transfer (smFRET) method to probe the distance between the pore and S346 (Fig. 4a). α3em and βem was engineered to produce α3_FRET_β_FRET_ GlyR, with and without S346E mutation, which accept cysteine-reactive chemical dyes only at α3C358 as the acceptor (LD655), and A1-tag-reactive^52,53^ dyes between α3S380 and P381 as the donor (LD555, see methods for details). α3C358 is close to the phosphorylation site S346, and α3S380 is at the intracellular terminus of helix M4, next to the pore. α3_FRET_β_FRET_ GlyR was immobilized on glass substrate by anchoring the GFP in β subunit M3/M4 loop, mimicking how GlyRs are anchored at post-synaptic densities^32,33,54^. FRET values in this setting (Fig. 4a) reflect on the distance between the phosphorylation site and the ion conduction pore.

smFRET measurements suggest structural flexibility in unphosphorylated (α3_FRET_β_FRET_ GlyR) M3/M4 loop, which is marginally affect by glycine activation or 2, 6-DTBP binding (Fig. 2b, d and Extended Data Fig. 7a). Unlike systems with more defined structural states where beautifully discrete FRET states and transitions were observed^55,56^, the FRET signals here appeared more unstable with frequent transitions of non-uniform magnitude, regardless of whether glycine was present (Fig. 4b, top and middle panels, Extended Data Fig. 7a, c). Histograms of average FRET values from multiple detections (apo: n = 184, with glycine: n = 219) shows very similar distributions that decomposes reasonably well to two Gaussians (see methods): one centered around 0.2 FRET value (peak 1) and the other around 0.4 (peak 2), with ~ 75% of population in peak1. Addition of 2, 6-DTBP (n = 247) had small effect in shifting peak 2 to ~ 0.5 FRET value, without affecting population distribution (Extended Data Table 2).

S346E mutation resulted in higher FRET values, which are further increased after application of 2, 6-DTBP (Fig. 4c, e, Extended Data Fig. 7b). FRET values of α3_FRET_S346Eβ_FRET_ GlyR, both in apo and in the presence of glycine, can be decomposed into two populations: one centered at ~ 0.3 FRET with 40% counts and anther broader peak centered at ~ 0.5 FRET with ~ 60% (Fig. 4e top and middle panels, Extended Data Table 2). These FRET values are significantly larger compared to without S346E mutation, suggesting a more compact M3/M4 loop conformation that brings the phosphorylation site closer to the ion conduction pore. The addition of 2, 6-DTBP dramatically increased FRET, resulting in ~ 67% population with ~ 0.5 FRET and ~ 33% with 0.75 FRET (Fig. 4e bottom panel, Extended Data Table 2), suggesting further compaction of M3/M4 loop towards the pore. That 2, 6-DTBP only increased phosphorylated α3β GlyR activity (S346E, Fig. 1f) coincides with the above measurements where FRET increase is much more evident with the phosphorylation mimic S346E (Fig. 4d, e).

### Increased homogeneity in M3/M4 loop distances upon phosphorylation and 2, 6-DTBP binding

**Figure 5:**
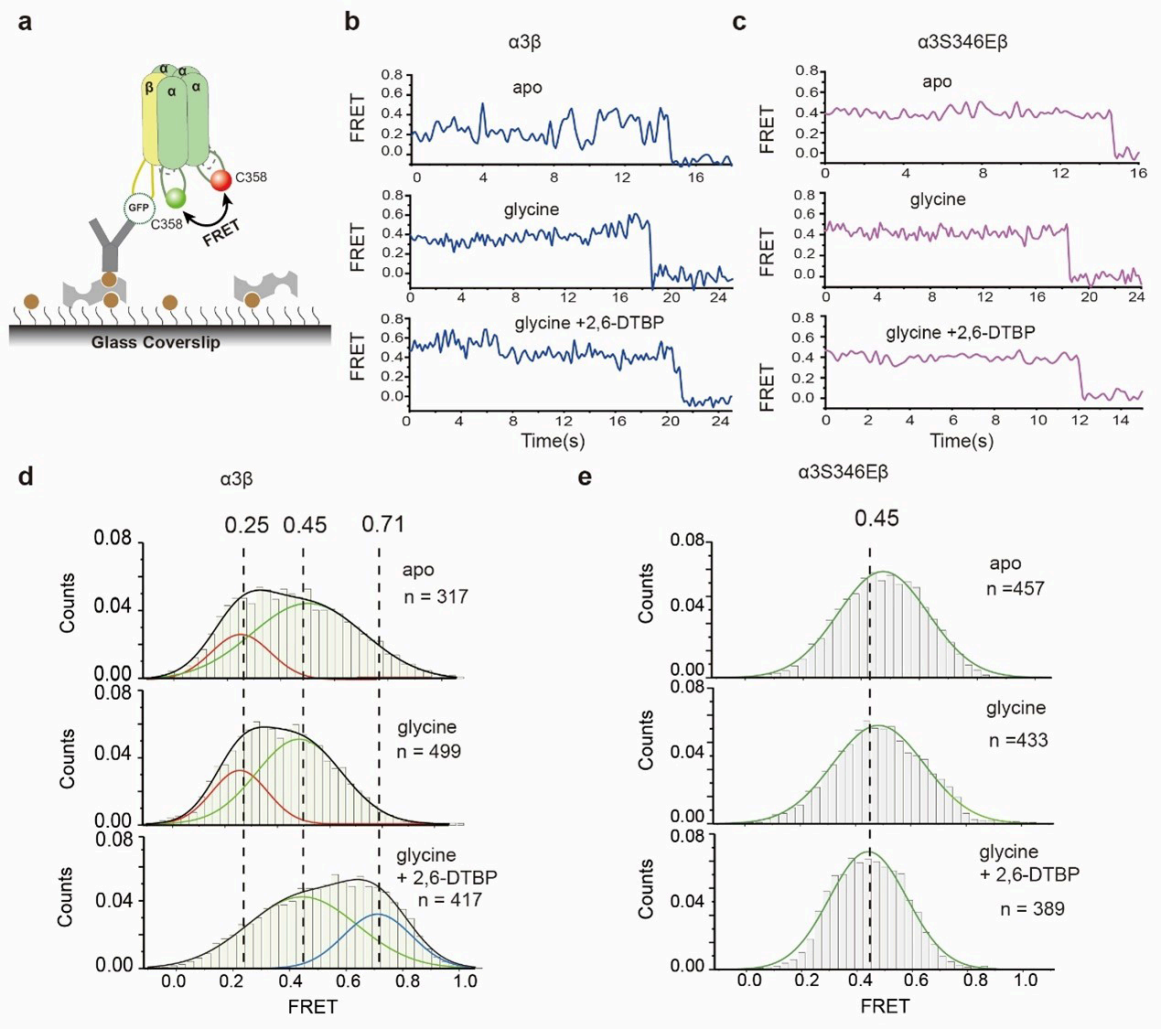
α3S346E mutation and 2, 6-DTBP affect the distances of M3/M4 loops between different α3 subunits **a,** Schematic of single-molecule FRET experiments for measuring the FRET values changes of M3/M4 loops between different α3 subunits. **b, c,** Representative single-molecule FRET time trace of α3β(b) and α3S346Eβ(c) GlyRs in apo (top), in presence of glycine (middle) and in presence of glycine and 2, 6-DTBP (bottom). **d, e,** Histograms of smFRET efficiency values from single-molecules traces for α3β(d) and α3S346Eβ(e) GlyR in apo (top), in presence of glycine (middle) and in presence of glycine and 2, 6-DTBP (bottom). Two smFRET distributions indicated by curves of Gaussian fitting and the sum of all distributions are shown as black lines.

To characterize whether the distance between M3/M4 loops from different α3 subunits is modulated by phosphorylation and 2, 6-DTBP binding, we measured FRET efficiencies between C358 of different α3 subunits (Fig. 5a, see methods for details).

Phosphorylation leads to more homogeneous inter-M3/M4 loop distances. Without phosphorylation (α3_FRET_β_FRET_ GlyR), two major populations were found with one peak centered at ~ 0.25 FRET value (24% counts in apo, n = 317; 28% glycine bound, n = 499) and the other centered at ~ 0.45 (76% apo, 72% glycine bound) irrespective of glycine binding (Fig. 5b, d upper and middle panel, Extended Data Table 3). After phosphorylation (α3_FRET_S346Eβ_FRET_ GlyR), a single component was identified centering at ~ 0.5 FRET, also independent of glycine binding (Fig. 5c, e upper and middle panel, apo n = 457, glycine bound n = 433, Extended Data Table 3). Coincidentally, the fluctuation of FRET values with respect to time becomes less prominent (Fig. 5c), suggesting a more stable spatial arrangement.

2, 6-DTBP showed a phosphorylation-dependent effect on inter-M3/M4 loop distances. Without phosphorylation, 2, 6-DTBP shifted FRET distribution from ~ 25% at ~ 0.25 FRET and ~ 75% at ~ 0.5, to ~ 68% ~ 0.44 FRET and ~ 32% at 0.71 (Fig. 5d lower, Extended Data Table 3, n = 417), suggesting increased overall compactness and the emergence of a more compact population. After phosphorylation, instead of increasing FRET values, 2, 6-DTBP binding showed a more subtle effect: it further increased the homogeneity of distance distribution, resulting in a narrower peak in histogram (Fig. 5e lower, n = 389). This seemingly inverse correlation between functional effect and loop conformational change hints at the working mechanism of 2, 6-DTBP, and will be discussed later.

## Discussion

With engineered α3β GlyR, we resolved structures of the human α3β GlyR before and after phosphorylation (mimic) of the large internal M3/M4 loop, as well as after 2, 6-DTBP potentiation (Fig. 2). Comparison of these structures point to a mechanism where phosphorylation and 2, 6-DTBP regulate ion conduction by changing the conformation of TM in an asymmetric manner (Fig. 3), coinciding with recently proposed asymmetric gating mechanism of heteromeric GlyR^5^. We further show, using smFRET, that phosphorylation leads to compaction of the M3/M4 loop towards the pore, and 2, 6-DTBP binding causes further compaction (Fig. 4), suggesting localized loop-TM interaction underlying TM conformational changes. In addition, we found that 2, 6-DTBP modulates inter-subunit M3/M4 loop distances in a phosphorylation-dependent manner (Fig. 5).

**Figure 6:**
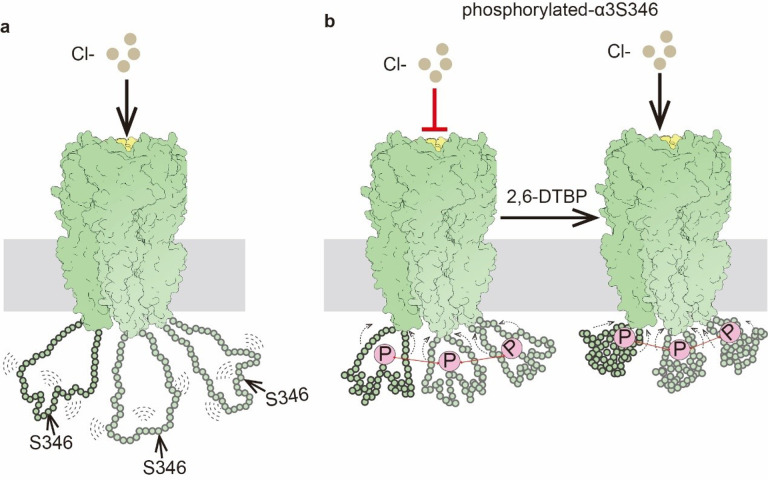
Proposed mechanism for α3 M3/M4 loop phosphorylation and 2, 6-DTBP modulation α3β GlyR activity **a,** During the entire functional cycle of non-phosphorylated α3β GlyR (close, open, and desensitized states), the M3/M4 loops of α3 subunits are very flexible and unstable. **b,** Left: Upon α3S346 is phosphorylated, the conformation of M3/M4 loop will change in two aspects: 1) the M3/M4 loop will be folding closer to the TMD; 2) the relative horizontal distances among M3/M4 loops will be fixed. These M3/M4 loops conformational changes result in pore conformational changes to decrease the influx of Cl^−^. Right: 2, 6-DTBP modulate the phosphorylated M3/M4 loop to fold and further closer TMD, leading to pore conformational changes and the phosphorylated α3β GlyR’s activity restoring.

Our findings suggest an underlying mechanism of how post-translational modification in the M3/M4 loop regulates α3β GlyR activity (Fig. 6), which is unlikely through altering the glycine binding pocket^38^ since no appreciable difference was identified in our structures (Extended Data Figure 6). The M3/M4 loop is randomly positioned below the intracellular pore in non-phosphorylated state (Fig. 6a). Once phosphorylated, the addition of negative charges likely introduces polar interactions within each loop, as well as between loops from different α3 subunits. These interactions lead to a more compact loop conformation with more homogenous distances between loops (Fig. 5e), and reduced distances between the phosphorylation site and the ion conduction pore (Fig. 4e), likely resulting in local accumulation of negative charge and decreased Cl^−^ conduction (Fig. 1e, Fig 6b), explaining how a distal phosphorylation decreases single channel conductance^41^. Changes in loop conformation also affect TM arrangement (Fig. 2f, g and Fig. 3b, e, h), but not sufficient to alter functional states and thus has minimal effects in gating^39,41^. The binding of 2, 6-DTBP causes further compaction of the phosphorylated loop toward the ion conduction pore (Fig. 4e, Fig. 6c). Such a dramatic change favors a more expanded TM configuration (Fig. 2 h, i and Fig. 3c, f, i), resulting in altered conduction pore geometry, increasing both unitary conductance and open probability^39,41,45^. We cannot rule out, at present, that the non-protein densities at widened inter-subunit interface (Fig. 3) may be (partly) arising from the M3/M4 loop.

2, 6-DTBP likely elicits function through interaction with M3/M4 loop. Although the density of 2, 6-DTBP cannot be unambiguously identified in our map reconstructions, several lines of evidence suggest interaction between 2, 6-DTBP with the M3/M4 loop. Despite having no functional effect when applied to non-phosphorylated GlyR^39,45^ (Fig. 1f), 2, 6-DTBP clearly changed the distribution of inter-loop distances (Fig. 5d). This suggests that the 2, 6-DTBP-induced conformational change originates from the M3/M4 loop, which leads to functional effects only when sufficient change propagates to the TM. This is consistent with 2, 6-DTBP causing loop compaction toward the conduction pore only for phosphorylated GlyR (Fig. 4d, e).

The of M3/M4 loop regulation mechanism proposed here implies localized interaction within one pentameric GlyR. Only when conformational changes of M3/M4 loop are sufficient to induce TM rearrangements, or cause local electrostatic potential change, functional effects arise. Apparently, whether phosphorylation-dependent interactions with unidentified players, or between different GlyRs play a role remain unclear. In addition, whether such mechanism is universal among Cys-loop receptors and other ion channels requires further investigation.

## Methods

### Plasmid constructs

The human glycine receptor α3 (NCBI: NP_006520.2), β (NCBI: NP_000815.1) and EP_2_(NCBI: NP_000947.2) sequence were amplified from cDNA clones (McDermott Center, UT Southwestern Medical center). The α3em sequence was generated by deletion 9 amnio acids of M3/M4 loop (residues A329-S337). α3S346Eem is the addition of S346E mutation site based on α3em. For the βem construct, we used the previously described βem construct^3,5^. M3/M4 loop (residues N334-N377) was replaced by GGSSAAA-monomeric enhanced green fluorescent protein (EGFP)-SGSGSG. A PA-tag (GVAMPGAEDDVV) and PreScission Protease site (LEVLFQ/GP) were inserted following signal peptide. The α3em and α3wt sequence were subcloned into BacMam expression vector^57^. The β wild type sequence were introduced into pLVX-IRES-ZsGreen1 vector (Clonetech) for electrophysiology. The human prostaglandin E2 receptor EP_2_ sequence was inserted into pLVX-IRES-mCherry vector (Clonetech) for electrophysiology. We designed constructs of α3_FRET_, α3S346E_FRET_ and β_FRET_ based on α3em, α3S346Eem, and βem for smFRET imaging, respectively. α3_FRET_ and α3S346E_FRET_ introduced a mutation C41V and inserted an A1 tag (GDSLDMLEWSLM) between α3S380 and α3P381. β_FRET_ introduced a mutation C115S. All mutations were introduced using sites-directed mutagenesis.

### Protein expression

Protein α3β and α3S346Eβ was expressed as described before^4,5^. The α3em, α3S346Eem, α3_FRET_, α3S346E_FRET_, βem and β_FRET_ plasmids were transformed into DH10BacY competent cells (Geneva Biotech) to produce bacmids. The bacmids were transfected into Sf9 cells (ATCC, CRL-1711) to generate baculovirus and then recombinant baculovirus titers were measured. Virus was added at MOI (multiplicity of infection) of 2 (at 3βem:1α3em ratio) to HEK293S GnTI^−^ cells (ATCC, CRL-3022) at a density of 2.5×10^6^ cells/ml. 10 mM sodium butyrate was added, and culture temperature was turned to 30 °C after transduction 12h. Cells were collected after induction 60h by centrifugation at 30,000 g for 20 minutes at 4 °C and stored at −80 °C until further use.

### Protein α3β GlyR purification and saposin nanodisc reconstitution for Cryo-EM data collection

Cell pellets were thawed and resuspended in lysis buffer (40 mM Tris pH 8.0, 50 mM NaCl, 2 mM MgCl_2_, 1 mM CaCl_2_, 2 mM Glycine, 20 μg/ml Dnase, 2 μg/ml leupeptin, 2 μM pepstatin, 0.8 μM aprotinin, 0.2 mM PMSF) rotated at 4 °C for 30 min under constant stirring, then cell debris was collected by centrifugation at 40,000g for 20 min. The cell debris was dounced and centrifugated at 40,000g at 4 °C for 20 min. The pellets were further homogenized and solubilized with buffer A (40 mM Tris pH 8.0, 200 mM NaCl, 2 mM MgCl_2_, 1 mM CaCl_2_, 2 mM Glycine, 20 μg/ml DNase, 2 μg/ml leupeptin, 2 μM pepstatin, 0.8 μM aprotinin, 0.2 mM PMSF, 0.75% (w/v) DDM, 0.075% (w/v) CHS and 0.075% (w/v) Na Cholate) for 40 min at 4 °C. Supernatant was collected from solubilized membranes by centrifugation at 40,000g for 30 min, and then PA-tag antibody (NZ-1) resin^58^ added to supernatant. The resin was collected and washed with 10 CV buffer B (20 mM Tris pH 8.0, 200 mM NaCl, 2 mM MgCl_2_, 1 mM CaCl_2_, 2 mM Glycine, 0.2 mM PMSF, 0.05% (w/v) DDM (Anatrace), 0.005% (w/v) CHS (Anatrace), 0.001% (w/v) Na Cholate (Anatrace)). Then resin bound with protein were mixed with PreScission protease (1:30 v/v) at RT for 1h to cleave PA tag. The flow through was collected, and resin were washed with another 2 CV buffer B. All proteins were pooled and concentrated to load onto Superose6 increase 10/300 GL column (GE Healthcare) in SEC buffer (20 mM Tris pH8.0, 200 mM NaCl, 2 mM Glycine, 0.05%(w/v) DDM, 0.005% CHS). Reconstitution of α3β GlyR into saposin nanodisc was modified from the published protocol^5^. 1:30:200 molar ratio of α3β: saposin: brain polar lipids extract (BPE) (Avanti) was used. α3β GlyR protein mixed with BPE at room temperature (RT) for 10 min. Saposin protein was added and the mixture was put at RT for another 2 min. The mixture was diluted with buffer (20 mM Tris pH 8.0, 200 mM NaCl, 2 mM Glycine) and incubate on ice for 30 min. Then bio-beads SM-2 (Bio-Rad) were added to the mixture and rotated overnight at 4 °C. After 12h, old bio-beads were removed and the fresh bio-beads were added for another 10h. The mixture was centrifuged for 30min at 4 °C before loading onto Superose 6 increase size exclusion column in SEC buffer (20 mM Tris-HCl pH 8.0, 200 mM NaCl, 2 mM glycine).

### Protein (α3β and α3S346Eβ GlyRs) purification in digitonin for Cryo-EM data collection

Cell lysis and protein solubilization by detergent follow the similar protocol as the protein purification for saposin nanodisc reconstitution. Briefly, solubilized membranes were cleared by centrifugation at 40,000g for 30 min. Supernatant was collected and added to PA-tag antibody (NZ-1) resin at RT. The resin was collected and washed with 5 CV buffer B and 5CV buffer C (20 mM Tris pH 8.0, 200 mM NaCl, 2mM MgCl_2_, 1 mM CaCl_2_, 2 mM Glycine, 0.06% (w/v) digitonin (Sigma-Aldrich)). Then, beads were mixed with PreScission protease (1:30 v/v) to cleave PA tag at RT for 1h. The resin was collected to get flow through, then resin was washed another 2 CV buffer C. Flow through and 2CV washed buffer C were pooled and concentrated to load onto Superose6 increase 10/300 GL column in SEC buffer (20 mM Tris-HCl pH8.0, 200 mM NaCl, 2 mM Glycine, 0.06%(w/v) digitonin). Good peak fractions were collected and concentrated to 6 mg/ml for grids freeze. For the sample with 2, 6-DTBP the buffer used throughout the purification process contained 500 μM 2, 6-DTBP and another 500 μM 2, 6-DTBP was added to cryo-EM sample for 1h before grid freezing.

### Cryo-EM sample preparation, data collection and image processing

3 mM final concentration (1 × CMC) of Fluorinated fos-choline 8 (Anatrace) was added into cryo-EM sample immediately before freezing. Grids (Quantifoil R1.2/1.3 400-mesh Au holey carbon grid) were glow-discharged. An FEI Vitrobot Mark IV Vitrobot (Thermo Fisher) was used to plunge freeze the grids after application of 3 μl sample at 4°C under 100% humidity.

Micrographs were collected using a Titan Krios microscope (Thermo Fisher) with a K3 Summit direct electron detector (Gatan) operating at 300 kV using the SerialEM data acquisition software. The GIF-Quantum energy filter was set to a slit width of 20 eV. Images were recorded with the pixel size of 0.415 Å in the super-resolution counting mode. Micrographs were dose-fractioned into 50 frames with a dose rate of 1.4 e^−^/Å/frame.

2-fold binning (0.83 Å pixel size after binning), motion correction and dose weighting of the movie frames were carried out using the Motioncorr2 program^59^. CTF correction was carried out using the CTFFIND 4 program^60^. The following image processing steps were performed in RELION 4^61^. Particles were initially picked using the Laplacian-of-Gaussian blobs and subjected to 2D classification to obtain good class-averages. Then good 2D classes were used as template for reference-based auto picking. Resulting particles were extracted with 4-fold binning for a further round of 2D classification. Good 2D class-averages were selected and subjected to 3D classification using an initial model downloaded from EMDB database (EMD-23148)^3^. For the α3β-gly GlyR in digitonin sample, 1 out of 6 classes in 3D classification appeared with good density for the entire channel (Extended Data Fig.2b). A single density blob for GFP was identified for the heteromeric α3β GlyR in digitonin sample. The density arising from GFP fusion on the βem subunit served as fiducial marker to differentiate the β subunit from the structurally similar α subunits. A further 3D classification into 4 classes with non-binned particles (0.83 Å pixel size) without particle alignment was performed. Partial signal subtraction^62^ was carried out to focus on the TMD. 1 indistinguishable good class resulted in a final of 19,993 particles. After reverting particles to un-subtracted version, CTF refinement, Bayesian polishing in RELION and non-uniform refinement^63^ in cryoSPARC^64^, an overall resolution of 3.8 Å was achieved, with local resolutions exceeding 3.5 Å in many regions (Extended Data Fig. 2c, d, e). For the α3β-gly GlyR in nanodisc sample, 1 out of 3 classes in 3D classification appeared with good density for the entire channel (Extended Data Fig.2g). A single density blob for GFP was identified for the heteromeric α3β-gly GlyR in nanodisc sample. A further 3D classification into 3 classes with non-binned particles (0.83 Å pixel size) without particle alignment was performed. 1 indistinguishable good class resulted in a final of 40,868 particles. After reverting particles to un-subtracted version, CTF refinement, Bayesian polishing in RELION and non-uniform refinement in cryoSPARC, an overall resolution of 3.8 Å was achieved, with local resolutions exceeding 3.5 Å in many regions (Extended Data Fig. 2h, i, j). For the α3S346Eβ-gly GlyR in digitonin, 1 out of 4 classes in 3D classification appeared with good density for the entire channel (Extended Data Fig.3b). A single density blob for GFP was identified for the heteromeric α3S346Eβ-gly GlyR sample. A further 3D classification into 4 classes with non-binned particles (0.83 Å pixel size) without particle alignment was performed. 1 indistinguishable good class resulted in a final of 9,628 particles. After reverting particles to un-subtracted version, CTF refinement, Bayesian polishing in RELION and non-uniform refinement in cryoSPARC, an overall resolution of 3.7 Å was achieved, with local resolutions exceeding 3.0 Å in many regions (Extended Data Fig. 3c, d, e).

For the α3S346Eβ-gly/2, 6-DTBP GlyR in digitonin sample, 1 out of 6 classes in 3D classification appeared with good density for the entire channel (Extended Data Fig.3g). A single density blob for GFP was identified for the heteromeric GlyR α3S346Eβ-gly/2, 6-DTBP GlyR sample. A further 3D classification into 4 classes with non-binned particles (0.83 Å pixel size) without particle alignment was performed. 1 indistinguishable good class resulted in a final of 22,755 particles. After reverting particles to un-subtracted version, CTF refinement, Bayesian polishing in RELION and non-uniform refinement in cryoSPARC, an overall resolution of 3.6 Å was achieved, with local resolutions exceeding 2.5 Å in many regions (Extended Data Fig. 3h, i, j). Resolutions were estimated by applying a soft mask around the protein densities with the Fourier Shell Correlation (FCS) 0.143 criterion. Local resolutions were calculated using Resmap^65^.

### Model building and refinement

Models of α3β-gly (in digitonin and nanodisc) and α3S346Eβ-gly GlyRs were bulit by fitting the structure of heteromeric human α1β desensitized state (PDB ID: 8DN4)^5^ into the Cryo-EM density maps of α3β-gly (in digitonin and nanodisc) and α3S346Eβ-gly GlyRs using Chimera^66^ and Coot^67^. Model of α3S346Eβ-gly/2, 6-DTBP GlyR was bulit by fitting the structure of heteromeric human α1β expanded-open state (PDB ID: 8DN2)^5^ into the Cryo-EM density map of α3S346Eβ-gly/2, 6-DTBP using Chimera^66^ and Coot^67^. The atomic model was manually adjusted in Coot. The final models were refined with real-space refinement module and validated with comprehensive validation module in PHENIX package^68,69^. Fourier shell correlation (FSC) curves were calculated between refined atomic model and the work/free half maps as well as the full map to assess the correlation between the model and density map. Statistics of cryo-EM data processing and model refinement are listed in Extended Data Table 1. Pore radii were calculated using the HOLE program^70^. Figures were prepared in UCSF Chimera^66^, ChimeraX^71^, and PyMOL^72^.

The final model of α3β-gly in nanodisc contained the α3 and β subunit amino acids except the following: α3 subunit of chain A (total 422aa, 349aa built, 73aa not built) A1-P7, K312-F328, D338-D382 and Q428 - D431; α3 subunit of chain B (total 422aa, 340aa built, 82aa not built) A1-M8, H311-F328, D338-D382 and H423-D431; α3 subunit of chain C (total 422aa, 345aa built, 77aa not built) A1-A6, H311-F327, D338-D382 and H423-D431; α3 subunit of chain D (total 422aa, 342aa built, 25aa not built) A1-M8, H311-F327, D338-M384, H427-D431. β subunit (total 444aa, 348aa built, 74aa not built) K1-R28, GSSAAA-EGFP-SGSGSG insertion and V378-P442.

The final model of α3β-gly in digitonin contained amino acids except the following: α3 subunit of chain A (total 422aa, 345aa built, 77aa not built) A1-M8, E313-F327, D338-R384 and H427-D431; α3 subunit of chain B (total 422aa, 340aa built, 82aa not built) A1-M8, K311-F327, D338-R384 and H423-D431; α3 subunit of chain C (total 422aa, 343aa built,79aa not built) A1-M8, E313-F327, D338-R384 and H423-D431; α3 subunit of chain D (total 422aa, 343aa built, 79aa not built) A1-M8, H311-F327, D338-K386 and H427-D431. The model of β subunit forα3β-gly in digitonin is the same as α3β GlyR-gly in nanodisc.

The final model of α3S346Eβ-gly contained amino acids except the following: α3 subunit of chain A (total 422aa, 343aa built, 79aa not built) A1-P7,H311-F327, D338-R385 and D425-D431; α3 subunit of chain B (total 422aa, 331aa built, 81aa not built) A1-P7, H311-F327, D338-R385 and H423-D431; α3 subunit of chain C (total 422aa, 336aa built, 31aa not built) A1-P7, H311-F327, D338-R385 and H423-D431; α3 subunit of chain D (total 422aa, 342aa built, 80aa not built) A1-P7, H311-F327, D338-R385 and H423-D431. The model of β subunit of α3S346Eβ-gly is the same as α3β GlyR-gly in nanodisc.

The final model of α3S346Eβ-gly/2, 6-DTBP contained the α3 and β subunit amino acids except the following: α3 subunit of chain A (total 422aa, 344aa built, 78aa not built) A1-M8, K312-F327, D338-R385 and I426-D431; α3 subunit of chain B (total 422aa, 334aa built, 81aa not built) A1-M8, K312-F327, D338-R385 and H423-D431; α3 subunit of chain C (total 422aa, 341aa built, 83aa not built) A1-P7, H311-F327, D338-R385 and H423-D431; α3 subunit of chain D (total 422aa, 345aa built, 79aa not built) A1-P7, H311-F327, D338-R385 and H427-D431. The model of α3S346Eβ-gly/2, 6-DTBP β subunit is the same as α3β GlyR-gly in nanodisc.

### Fluorescence-Detection Size-Exclusion Chromatography (FSEC) expression assay

Fluorescence was detected using the RF-20Axs fluorescence detector for HPLC (Shimadzu, Japan) (GFP excitation: 480 nm, emission: 512 nm) as EGFP was fused into βem construct for FSEC assay. 2 μl of Lipofectamine 3000 (Thermo Fisher Scientific, US) mixing with 1 μg of plasmid (at 1α3:3β ratio) was transfected into HEK293T cells for 12 well plate each well. Cells were incubated in a CO_2_ incubator (37 °C, 8% CO_2_) for 48 h after transfection and solubilized with 50 μl buffer B for 1 h. After centrifugation (40,000 g, 30 min), 50 μl of the sample was applied to a Superose 6 Increase 10/300 GL column (GE Healthcare) equilibrated with buffer D (20 mM Tris pH 8.0, 200 mM NaCl, 2 mM glycine, 0.025%DDM) for the FSEC assay.

### Whole cell patch clamp

The glycine EC_50_ values were measured on α3β GlyR and α3S346Eβ GlyR expressed in HEK293T cells (ATCC, CRL-3216). Plasmids were transiently transfected using Lipofectamine 3000 reagent (Invitrogen). Total 0.8 μg of DNA was transfected at 1α3:3β ratios for 35 mm dish. Whole-cell recordings were made after 17–24h transfected at 22 °C. GFP fluorescence was used to identify the cells expressing the heteromeric α3β and α3S346Eβ GlyRs. The bath solution contained (in mM): 10 HEPES-NaOH pH 7.4, 10 KCl, 125 NaCl, 2 MgCl_2_, 1 CaCl_2_ and 10 glucoses. The pipette solution contained (in mM): 10 HEPES-NaOH pH 7.4, 150 KCl, 5 NaCl, 2 MgCl_2_, 1 CaCl_2_ and 5 EGTA. The resistance of borosilicate glass pipettes between 2~7 MΩ. The voltage held at −70 mV and a Digidata 1550B digitizer (Molecular Devices) was connected to an Axopatch 200B amplifier (Molecular Devices) for data acquisition. Analog signals were filtered at 1 kHz and subsequently sampled at 20 kHz and stored on a computer running pClamp 10.5 software. Data analysis was performed by Origin 2018 software (Origin Lab). Hill1 equation was used to fit the dose-response data and derive the EC_50_ (*k*) and Hill coefficient (*n*). For glycine dose response experiment, we fit the data using equation $I=I_{0}+\left(I_{\text{max}}-I_{0}\right)\frac{x^{n}}{k^{n}+x^{n^{’}}}$, where *I* is current, *I*_*0*_ is the basal current (accounting mostly for leak, very close to 0), *Imax* is the maximum current and *x* is glycine concentration. All start point is fixed at 0 during fit. Measurements were from 7–11 cells, mean and S.E.M. values were calculated for each data point.

For experiments of PGE_2_ modulation GlyR, total 1 μg of plasmid (0.6 μg GlyR at 1α3:3β ratios and 0.4 μg EP_2_) was transfected for 35 mm dish. Whole-cell recordings were made after 17–24h transfected at 22 °C. Both in presence of GFP (GlyR) and mCherry (EP_2_) fluorescences were used to identify the cells co-expressing the heteromeric α3β GlyR and EP_2_ receptor. PGE_2_ (10 μM concentration used) was applied by perfusion system at a rate of 1–2 ml/min. At least 5 times current response evoked by 1 mM glycine was recorded before application of PGE_2_. After application PGE_2_ for about 2 minutes, the currents reached steady state. This steady state of inhibition kept another 3 min with PGE_2_ application. Then bath solution without PGE_2_ was applied to wash out.

2, 6-DTBP (100 μM concentration used) was also applied by perfusion system at a rate of 1–2 ml/min. After 3 to 5 times current response evoked by 30 μM glycine of baseline recording, 2, 6-DTBP (100 μM) was applied to bath solution for 4–6 min until the currents increase reaching saturation. The increase in current is recorded every 40 seconds.

### Protein purification and labeling for smFRET

Cell lysis and protein solubilization by detergent follow the protocol as the protein purification for saposin nanodisc reconstitution excepting that 20 mM HEPES-NaOH, pH7.4 was used instead of 20 mM Tris-HCl, pH8.0. Peak fractions of protein were collected and concentrated to 1 mg/ml. α_FRET_β_FRET_ was equally divided into two parts. One part protein was labeled with CoA-LD555 and LD655-MAL.The protocol as described below: 10 μM TCEP was added to protein then incubated for 30 min on ice. α_FRET_β_FRET_ was labeled first by incubating protein with LD655-MAL at 1:3 (protein: LD655-MAL) molar ratio at 4 °C for overnight in the dark. α_FRET_β_FRET_ was labeled further by incubating protein with 20 μM AcpS, 10 μM CoA-LD555, 20 mM MgCl_2_, 20 mM HEPES-Na, pH 7.4 at RT for 4h protecting from light. Another part of the protein was labeled with LD555-MAL and LD655-MAL in the dark at 1:3:3 molar ratio (protein: LD555-MAL: LD655-MAL). To remove free dye, the solution with labelled protein was then loaded onto PD-10 desalting column (GE Healthcare) equilibrated in the buffer E (20 mM HEPES-NaOH, pH7.4, 200 mM NaCl, 0.03% (w/v) DDM, 0.003% (w/v) CHS), and the resulting flow-through was loaded onto a second desalting column equilibrated in buffer E. The flow through containing pure labeled protein was centrifuged at 18,000g for 1 h at 4 ° C to remove insoluble aggregates. FRET-Labeled α_FRET_β_FRET_ were aliquoted and frozen at −80 °C, and freshly thawed before the experiments.

### Glass slides preparation for smFRET imaging

The glass slides are cleaned by soaking for 1.5 h at room temperature in piranha solution (≥98% H_2_SO_4_ and 30% H_2_O_2_ in a 3:1 ratio) in jugs. The procedure is carried out in a hood. The glass slides were sonicated for 3 times for 10s/time (once at the started soaking, once at 45 min and once at the end) and the washed with ddH_2_O for 5 times in jug. Then the treated glass slides are further soaked in 1M KOH for another 30 min and washed for 5 times using running ddH_2_O. During soaking with KOH, the glass slides were sonicated for another 3 times for 1mim/time (once at the started soaking, once at 15 min and once at the end). After washing procedures, the glass slides are drained on air in a vertical position. The soaked glass slides were covered with 25% mPEG-sliane 5k (Sigma-Aldrich) with 1% Biotin mPEG-silane 5k (Sigma-Aldrich) at 90°C on met al plate covering by Petri dish for 30 min. Finally, the glass slides were washed with running ddH_2_O and then drained on air in a vertical position. Coated glass slides were stored at −20 °C until further use.

### TIRF-based single-molecule FRET imaging

For direct immobilization of α_FRET_β_FRET_, the imaging surface was first exposed to 0.2 μM NeutrAvidin (Thermo Fisher Scientific) and then 50 nM Biotin Anti-GFP antibody (abcam, ab6658) in buffer F (50 mM HEPES-NaOH, pH7.4, 150 mM NaCl). The surface was washed and exchanged into imaging buffer (50 mM HEPES-NaOH pH 7.4, 150 mM NaCl, 10 mM MgCl_2_, 0.8% (w/v) glucose). FRET-labeled GlyR variants was diluted to 0.7 nM and bound to a NeutrAvidin/ Biotin anti GFPab-coated glass slide surface for 30 min in imaging buffer with 2 μM 25-nucleotide DNA duplex (IDT) and 10 mg/ml BSA (Jackson Immunoresearch) as surface blocking agents. To measure smFRET in apo state, imaging was performed in imaging buffer. To measure the effect of glycine on smFRET value, imaging was performed in imaging buffer added 2mM glycine. To detect the modulating of 2, 6-DTBP on M3/M4 loop, imaging was performed in imaging buffer added 2 mM glycine and 500 μM 2, 6-DTBP and waiting for 30min before imaging recording. TIRF-based smFRET imaging experiments were performed at 22 °C with a custom-built TIRF microscope. Fluorescence emission from LD555 and LD655 was collected by a 60X, 1.27 NA water immersion objective (Leica), spectrally split in a MultiCam Device (Cairn) and collected with two synchronized Flash 4.0 V3 camera (C13440–20CU, Hamamatsu) with 2×2 pixel binning. SmFRET imaging recordings were performed by exciting with the Gem 560 nm laser (Laser Quantum) laser at 50 mW and acquiring 200 frames per movie at a 200 ms/frame rate in both donor and acceptor channels.

### Analysis of TIRF-based single-molecule data

Image movies were analyzed with Cornell SPARTAN version 3.7.0^73^ following manual. Molecules were detected as local intensity maxima in an image combing with donor and acceptor channels (aligned using the iterative closest points algorithm) averaged over the first 10 frames and background subtracted with threshold 100. The distances of molecules smaller than 3.5 pixels were excluded from analysis. Traces were extracted from the selected intensity maxima by summing the 9 most intense pixels for each fluorescence channel. Selected traces were saved for further analysis if they met the following criteria for experiments recorded with 200 ms (10 ms) time resolution: FRET lifetime > 5, donor acceptor correlation coefficient −1 to 0.5, signal-to-noise >8, #cy3 blinks<4 and remove overlapping traces. Saved races is then manually viewed and selected as all FRET section for further analysis according following criteria: Donor-acceptor fluorescence exchange time more than 5s (25 frames); Donor and acceptor fluorescence were found to bleach in a single step. Single-molecule traces showing dynamics before photobleaching. More than 180 typically molecules at each condition were manually selected, and FRET values for individual each conduction was accumulated in histograms. Histogram distributions were analyzed with a double Gaussian equation to reveal reoccurring mean FRET values using Origin 2018 software (OriginLab). The correlation results of Gaussian fitting analysis were listed on Extended Data Table 2 and 3. FRET histograms showed in results are averaged from the first 25 frames (total 5s).

### Plotting and statistics

Glycine dose-response curves fitting was used Origin 2018 software (OriginLab). Plotting for PGE_2_ and 2, 6-DTBP modulation GlyRs were carried out by GraphPad Prism (GraphPad Software). Plotting, distribution fitting and statistics for all single-molecule data were carried out using Origin 2018 (OriginLab). All errors represent the S.E.M.

## Data Availability

The density maps for the cryo-em data have been deposited in the Electern Microscopy Data bank under accession codes EMD-44754 (α3β-gly in nanodisc), EMD-44755 (α3β-gly in digitonin), EMD-44756 (α3S346Eβ-gly in digitonin), EMD-44763 (α3S346Eβ-gly/2, 6-DTBP in digitonin). The coordinates have been deposited in the Protein Data Bank under accession codes 9BOY (α3β-gly in nanodisc), 9BOZ (α3β-gly in digitonin), 9BPO (α3S346Eβ-gly in digitonin), 9BP7(α3S346Eβ-gly/2, 6-DTBP in digitonin).
